# Epilepsy and Cannabis: A Literature Review

**DOI:** 10.7759/cureus.3278

**Published:** 2018-09-10

**Authors:** Sidra Zaheer, Deepak Kumar, Muhammad T Khan, Pirthvi Raj Giyanwani, FNU Kiran

**Affiliations:** 1 Dow Medical College, Dow University of Health Sciences (DUHS), Karachi, PAK; 2 Internal Medicine, Jinnah Postgraduate Medical Centre, Karachi, PAK; 3 Neurology, Charleston Area Medical Center, Charleston, USA; 4 Civil Hospital, Dow University of Health Sciences (DUHS), Karachi, PAK; 5 Internal Medicine, Basic Health Unit Larhi, Gambat, Gambat, PAK

**Keywords:** epilepsy, cannabis, refractory epilepsy

## Abstract

Epilepsy is considered to be one of the most common non-communicable neurological diseases especially in low to middle-income countries. Approximately one-third of patients with epilepsy have seizures that are resistant to antiepileptic medications. Clinical trials for the treatment of medically refractory epilepsy have mostly focused on new drug treatments, and result in a significant portion of subjects whose seizures remain refractory to medication. The off-label use of cannabis sativa plant in treating seizures is known since ancient times. The active ingredients of this plant are delta-9-tetrahydrocannabinol (THC) and cannabidiol (CBD), the latter considered safer and more effective in treating seizures, and with less adverse psychotropic effects.

Clinical trials prior to two years ago have shown little to no significant effects of cannabis in reducing seizures. These trials seem to be underpowered, with a sample size less than 15. In contrast, more recent studies that have included over 100 participants showed that CBD use resulted in a significant reduction in seizure frequency. Adverse effects of CBD overall appear to be benign, while more concerning adverse effects (e.g., elevated liver enzymes) improve with continued CBD use or dose reduction.

In most of the trials, CBD is used in adjunct with epilepsy medication, therefore it remains to be determined whether CBD is itself antiepileptic or a potentiator of traditional antiepileptic medications. Future trials may evaluate the efficacy of CBD in treating seizures due to specific etiologies (e.g., post-traumatic, post-stroke, idiopathic).

## Introduction and background

Epilepsy is one of the most common non-communicable neurological diseases. Its incidence is 50.4 per 100,000 people per year and is more common in low to middle-income countries, i.e., 81.7 per 100,000 people per year [1,2]. About one-third of the patients suffering from epilepsy have drug-resistant epilepsy. A patient is said to have drug-resistant epilepsy if their seizures cannot be controlled even after using the appropriate dose of at least two antiepileptic medications. Drug-resistant epilepsy is associated with reduced quality of life, serious psychosocial consequences, and cognitive problems [3,4]. Therefore, a lot of research is being conducted to find the treatment of drug-resistant epilepsy.

Cannabis sativa is a plant that has been used to treat epilepsy, pain and anorexia since ancient times. It has more than 80 phytocannabinoids, most abundant among which are delta-9-tetrahydrocannabinol (THC) and Cannabidiol (CBD) [5]. The role of THC predominant preparations came into play in the 19th century and since the last couple of years, clinical trials on less controversial CBD started [6]. More recently trials on Propyl analog of CBD, Cannabidivarin (CBDV), have also started. The reason for developing and preferring CBD and CBDV as an anticonvulsant drug is because THC has many psychiatric side effects [7]. Although CBD is the most studied molecule for the treatment of epilepsy its mechanism of action is not fully understood until now [8,9].

## Review

Methodology

We carried this literature review in January 2018. The data were collected from the trials which were published during the year 1978 to 2017. Major search engines used were PubMed and Google Scholar. We used keywords such as cannabis, epilepsy and randomized control trials. We restricted our data collection to those randomized control trials which were published in the English language only.

We included six trials which were performed in the year 1978-2017. The inclusion criteria included the trials reporting a decrease in seizure frequency and were performed for at least 12 weeks. We did not include any case reports.

Results

There were six studies which met our inclusion criteria and were included in our literature review. In each trial, patients continued their regular antiepileptic drugs along with either placebo or CBD.

In 1978, Mechoulam and Carlini conducted a double-blinded clinical trial. A total of nine patients with treatment-resistant epilepsy were included. Out of those nine patients, four were randomly assigned in the CBD group and five in the placebo group. Patients in the CBD group were given 200 mg of CBD daily for three months. Two of the epilepsy patients had no seizure during the whole three months of treatment, and the third one had partial improvement while no improvement was observed in the fourth one. There was no significant toxicity [10].

In 1980, Cunha et al. conducted a double-blinded trial. They included 15 patients suffering from secondary generalized epilepsy with a temporal focus. Out of 15, eight patients were included in the treatment group and seven were included in the placebo group. Each patient in the treatment group received 200–300 mg of CBD daily for a period of eight to 18 weeks. Electroencephalography (EEG) and electrocardiography (ECG) were performed at 15–30 days interval. Four of the patients in the treatment group were seizure-free while three had partial improvement and the remaining one did not have any effect [11].

Ames et al. conducted a double-blinded clinical trial, in which 12 mentally retarded institutionalized patients with uncontrolled seizures were assigned in either the treatment or control group with six patients in each group. Patients in the treatment group were given 300 mg of CBD for one week and then 200 mg of CBD for the next three weeks. No difference in the seizure frequency was reported [12].

In Trembly et al., a crossover trial was conducted with 12 patients. In this trial, all 12 patients were treated with placebo for six months followed by 300 mg of CBD or placebo in a crossover trial, which lasted for an additional 12 months. There was no any change in seizure frequency and side effects [13].

Devinsky et al. included 162 patients with Dravet syndrome and Lennox-Gastaut syndrome in a safety analysis and a subgroup with 137 patients were included in the efficacy analysis. The patients were treated with 2–5 mg/kg of CBD per day which was titrated up to 50 mg/kg/day for a period of 12 weeks. Reduction in monthly seizure frequency in the efficacy group was 36.5%, with the greatest reduction recorded in patients with atonic and focal seizures. Most common adverse effects faced by patients in the safety analysis group were somnolence, decreased appetite, diarrhea, fatigue, and convulsions. The serious adverse effects were reported in 30% of the patients, including one death which was regarded as unrelated to the study drug. Status epilepticus was the most common serious adverse effect reported [5].

Devinsky et al. in the year 2017 conducted a double-blind clinical trial. In this trial, 120 children and young adults with Dravet syndrome and drug-resistant epilepsy were randomly assigned. Patients in the treatment group received 20 mg/kg/day for a period of 14 weeks including a titration phase of two weeks. The median frequency of seizures decreased from 12.4 to 5.9 in the treatment group and 14.9 to 14.1 in the placebo group. In the treatment group 43% of the patients and in the placebo group 27% of patients had >50% reduction in the seizure frequency. Patients having seizures without convulsions were not affected by the CBD therapy remarkably. In the treatment group, 5% of the patients also became seizure-free during treatment. However, 75% of the patients in the treatment group faced adverse effects such as diarrhea, vomiting, fatigue, pyrexia, and somnolence – somnolence being the most common (Table 1) [14].

**Table 1:** Summary of Results. CBD: Cannabidiol

Study	No. of Patients	Duration	Outcome	Side Effects
Mechoulam and Carlini (1978) [10]	Four in CBD group, Five in placebo group	Three months	Two were seizure-free, one had partial improvement, one did not have any effect	None
Cunha et al. (1980) [11]	Eight in CBD group, Seven in placebo group	Four and a half months	Four were seizure-free, three had partial improvement, one did not have any effect	Somnolence
Ames et al. (1986) [12]	Six in CBD group, Six in placebo group	CBD 300 mg/day x one week 200 mg/day x next three weeks	No difference	Somnolence
Trembly et al. (1990) [13]	10	12 months (randomized to either group for six months and then crossover on the alternative treatment)	No change in seizure frequency	None
Devinsky et al. (2016) [14]	137	12 weeks	Median reduction noted in monthly motor seizures was 36.5%	Somnolence, decreased appetite, diarrhea Serious adverse effects included status epilepticus in 6% of patients
Devinsky et al. (2017) [15]	61 in CBD group, 59 in placebo group	14 weeks	43% had a decrease in seizure frequency 5% were seizure free	Diarrhea, vomiting, fatigue, pyrexia, somnolence

Discussion

About one-third of epileptic patients do not respond well to the conventional antiepileptic drugs [5]. Moreover, there are many side effects associated with them such as osteomalacia and anemia. This demands a need for an antiepileptic drug in the market with better efficacy and lesser adverse effects. For centuries, CBD is considered by the general populace to have anticonvulsants properties. However, these substances could not find a place in the current prescription regimen to treat seizures because of the two main reasons. First, no sufficient number of trials have been done which could prove their efficacy in treating or preventing seizure episodes. Second, there are concerns about their safety in the long-run.

Endocannabinoids (cannabinoids synthesized normally within the central nervous system (CNS)) have a role in decreasing the release of excitatory neurotransmitter in CNS, hence preventing from seizures [15]. They act on CB1 and CB2 receptors with former being expressed by central and peripheral neurons, while the latter are mainly expressed by immune cells but are also found in the brain cells [16]. An old thought of CB1 receptors being the only ones involved into neuronal activity regulation has been challenged by a recent experimental study on rats, which concluded that loss of both types of receptors will result into a spontaneous and more severe form of seizures than the loss of CB1 receptors alone [17]. This formulates that the development of potential drugs enhancing the activity of these receptors could be used as a therapeutic means for seizure disorders. Cannabis is extracted from the Cannabis Sativa plant, which has more THC than CBD. THC is psychoactive while CBD has little to no psycho-activity. Also, CBD has more antiepileptic properties, thus drawing attention to the preparations with more CBD to THC proportion [18]. Lesser adverse effects with CBD could be there because CBD has a weak activity at CB1 and CB2 receptors. CBD instead works by other mechanisms such as transient receptor potential (TRP) cation channels resulting in a decrease in the presynaptic release of glutamate [8,19].

In 1977, in an experiment conducted on rats, it was postulated that the anticonvulsant effects of CBD could be compared with those of phenytoin, and protective effects to decrease relapses were comparable to phenobarbital. Short-term analysis has also shown that CBD is a safe drug in humans with no psychotropic activity, no changes on clinical and laboratory examination, and no effects on EEG and ECG [11]. Besides, having the long half-life (30 hours when given through the intravenous route and 23 hours when given orally) will also be helpful in patient-compliance [20].

Although studies had been conducted on CBD to appreciate its potential antiepileptic effects, many of them, until 2016, were carried out over a short duration of three to four months and remained underpowered with the maximum sample size of 15 patients. In 2016 and 2017, Devinsky et al. did trials to appreciate the effects of CBD on seizures in patients with childhood-onset seizures, especially with Dravet syndrome. More than 100 patient participants were in his studies. He observed a good antiepileptic effect of CBD with a few adverse effects. Most of them were mild to moderate and included somnolence, decreased appetite, fatigue, diarrhea, and increased convulsions, that showed CBD might also have pro-convulsive properties [5,14]. In some patients, a few abnormalities in the liver function tests were reported, which gradually returned to normal with the continuous use of CBD [14]. Whether CBD per se has anticonvulsant properties or it potentiates the effects of traditional antiepileptic drugs is not yet clear. CBD has been shown to increase the concentration of a few other antiepileptic drugs especially clobazam through its inhibitory action on cytochrome P450 system [21].

Recently, the first time a randomized, controlled, double-blinded multi-center study was done to appreciate the potential effects of CBD in controlling drop seizures in patients with Lennox-Gastaut syndrome (a childhood onset seizure disorder associated with encephalopathy which usually is multi-drug resistant) [22]. A total of 171 patients were randomly divided into a drug or placebo group in 1:1 pattern. There was 43.9% reduction of drop seizures in CBD group patients and 21.8% of the reduction in the patients who were in the placebo group. Besides drop seizures, the frequency of other types of seizures was also reduced drastically which reflected a wide spectrum of effects of CBD in controlling different types of seizures. The most common adverse effects (in >10% of cases), though mild to moderate in severity, including somnolence, diarrhea, and decreased appetite were seen in 86% of patients who were in CBD group and 69% of patients who were in the placebo group. The most serious treatment-related adverse effect occurred was an elevation of liver enzymes (alanine aminotransferase, aspartate aminotransferase, and gamma-glutamyltransferase) in >3% cases but resolved on their own with the continuation of the treatment. Additional studies on adverse effects of CBD are needed since most of the patients in the studies so far were concomitantly taking other antiepileptics such as valproate and clobazam, which may have confounded the results.

In 2017, an Australian Nationwide survey on medical cannabis use for epilepsy was carried out, which included 976 responders (patients with epilepsy and/or parents/guardians of patients with epilepsy). It showed that around 15% of patients used cannabis irrespective of their physician’s knowledge to control their multi-drug resistant seizures and to get rid of the adverse effects associated with traditional antiepileptic drugs. Most of them reported an improvement in their seizures [23].

## Conclusions

We have reviewed multiple clinical trials on drug-resistant epilepsy and have discussed their results in this review article. There is an increasing interest in developing cannabis preparations for the treatment of drug-resistant epilepsy as they are observed to be more efficacious with less side effect profile. Hence, we encourage research in this area in order to help decrease the morbidity and mortality associated with drug-resistant epilepsy.
